# Antibacterial Inhibitory Effects of *Punica Granatum* Gel on Cariogenic Bacteria: An *in vitro* Study

**DOI:** 10.5005/jp-journals-10005-1426

**Published:** 2017-06-01

**Authors:** Grazielle Millo, Apa Juntavee, Ariya Ratanathongkam, Natsajee Nualkaew, Jomjai Peerapattana, Supaporn Chatchiwiwattana

**Affiliations:** 1 Graduate Student, Department of Pediatric Dentistry, Faculty of Dentistry, Khon Kaen University, Khon Kaen, Thailand; 2 Associate Professor, Department of Pediatric Dentistry, Faculty of Dentistry, Khon Kaen University, Khon Kaen, Thailand; 3 Associate Professor, Department of Oral Biology, Faculty of Dentistry, Khon Kaen University, Khon Kaen, Thailand; 4 Assistant Professor, Department of Pharmacognosy and Toxicity, Faculty of Pharmaceutical Sciences, Khon Kaen University, Khon Kaen Thailand; 5 Associate Professor, Department of Pharmaceutical Technology, Faculty of Pharmaceutical Sciences, Khon Kaen University, Khon Kaen Thailand; 6 Associate Professor, Department of Oral Biology, Faculty of Dentistry, Khon Kaen University, Khon Kaen, Thailand

**Keywords:** Antibacterial, *Punica grantum* gel, Punicalagin, *Streptococcus mutans.*

## Abstract

**Aim:**

This study evaluated the *in vitro* antibacterial effects of the formulated *Punica granatum* (PG) gel against *Streptococcus mutans, Streptococcus sanguinis,* and *Lactobacillus casei.*

**Materials and methods:**

The PG extract was dissolved in water at 500 mg/mL. High performance liquid chromatography (HPLC) was used for identification and quantification of chemical marker punicalagin. Minimum bactericidal concentration (MBC) and time-kill assay (TKA) were investigated. Antibacterial activities of the formulated PG gel, 2% chlorhexidine (CHX) gel and blank gel were tested by measuring the zones of inhibition through agar well diffusion method.

**Results:**

The HPLC results showed presence of punicalagin at 2023.58 ± 25.29 pg/mL in the aqueous PG extract and at 0.234% (w/w) in the formulated PG gel. The MBC for *S. mutans, S. Sanguinis,* and *L. casei* were 250, 125, and 500 mg/mL respectively. The TKA of 500 mg/mL aqueous PG extract showed total inhibition of S. *mutans, S. Sanguinis,* and *L. casei* at 6, 1, and 24 hours contact time respectively. Agar well diffusion revealed that for S. *mutans,* CHX gel > PG gel > blank gel; for S. *sanguinis,* CHX gel = PG gel > blank gel; for *L. casei,* CHX gel > PG gel = blank gel. Comparison of the PG gel potency showed that S. *sanguinis* = S. *mutans* > *L. casei.*

**Conclusion:**

The PG gel equivalent to 0.234% punicalagin (w/w) inhibited *S. mutans* and *S. sanguinis* but not *L. casei* within 24 hours incubation period and has the potential to be used for caries prevention.

**How to cite this article:**

Millo G, Juntavee A, Ratanathongkam A, Nualkaew N, Peerapattana J, Chatchiwiwattana S. Antibacterial Inhibitory Effects of *Punica Granatum* Gel on Cariogenic Bacteria: An *in vitro* Study. Int J Clin Pediatr Dent 2017;10(2):152-157.

## INTRODUCTION

Dental caries has been identified as one of the most prevalent chronic conditions and is a major problem for children all over the world.^1^ Untreated caries in deciduous teeth was the 10th most prevalent medical condition, affecting 9% of the global population.^2^ Caries is characterized by an early acquisition and overgrowth of several species of cariogenic bacteria, such as *Streptococcus mutans, Streptococcus sanguinis,* and *Lactobacillus casei.*

*Streptococcus mutans,* the primary etiologic factor for dental caries, causes demineralization of inorganic tooth structure by metabolizing sucrose to lactic acid. It can also colonize tooth surfaces and initiate plaque formation through their ability to synthesize and bind extracellular polysaccharides (glucan) using the enzyme glucosyltrans-ferase.^3-5^ *Streptococcus sanguinis* initiates the aggregation of other oral bacteria and cause maturation of the dental plaque, while *L. casei* is commonly found in advanced caries lesions extending to dentin.^6-9^ Several antibiotics and antimicrobial agents, such as chlorhexidine (CHX) has been used to eliminate cariogenic bacteria from the oral flora. However, their clinical use is limited due to undesirable side effects including microorganism susceptibility, vomiting, diarrhea, and tooth staining.^10,11^ These problems stressed the importance of further research to develop alternative antibacterial agents from natural sources with focus on safety for humans and efficacy in the treatment and prevention of dental caries.

*Punica granatum* (PG), commonly known as pomegranate, is widely renowned for its variety of therapeutic effects and is relatively low-cost and locally available in most of the Southeast Asian countries.^12^ Pomegranate is also documented to have minimal toxicity and adverse effects.^13-15^ Studies have shown that PG has antimicrobial effects against different microorganisms.^16-18^ However, not enough information is available concerning the *in vitro* antimicrobial effect of a formulated PG gel against cariogenic microorganisms specifically *S. mutans, S. sanguinis,* and *L. casei.*

## AIM

This study aimed to evaluate and compare the *in vitro* antibacterial effects of the formulated PG gel against planktonic forms of cariogenic bacteria, such as *S. mutans, S. sanguinis,* and *L. casei.*

## MATERIALS AND METHODS

### Preparation of the PG Extract Sample

*Punica granatum* extract powder extracted from pomegranate pericarp (NuSci®, USA) was dissolved in distilled water at a 500 mg/mL concentration. The mixture was shaken in a vortex mixer for 1 minute and centrifuged at 10,000 × g for 10 minutes at 25°C. The supernatant was then filtered through a nylon filter with pore size 0.45 um prior to further testing and laboratory investigation.

### High Performance Liquid Chromatography

High Performance Liquid Chromatography (HPLC) analysis was carried out using Agilent 1100 series equipped with an Agilent 1100 series photodiode array detector and autosampler (Agilent Technologies, USA) with a reversed phase 4 mm internal diameter × 250 mm length, Kromasil 5 um 100A C18 column (Phenomenex, USA). The mobile phase consisted of solvent A: 0.1% trifluoroacetic acid (Sigma-Aldrich, USA) in water and solvent B: Methanol (Merck, Germany) with an elution profile modified from Madrigal-Carballo et al^19^: 100 to 73% solvent A for 30 minutes, 73 to 45% solvent A for the next 15 minutes, 45 to 0% solvent A for 5 minutes, maintained 0% solvent A for 10 minutes followed by a 10 minutes re-equilibration time back to 100% solvent A. Flow rate was set at 1 mL/minute and 20 μL of the sample was injected into the column per cycle. The UV detector was set at 254 nm at 25°C. Punicala-gin analytic standard of 98% purity (Sigma-Aldrich, USA) was extracted from pomegranate. A series of 25 to 200 μg/ mL punicalagin standard was diluted in water to make the calibration curve and method validation experiments.

### Bacterial Culture

Standard strains of *S. mutans* (DMST18777) and *S. sanguinis* (DMST18782) from National Institute of Health, Thailand were prepared and cultured separately in Todd Hewitt Broth (THB, Bacto^™^, USA) and Mitis Salivarius Agar (MSA) (Difco^™^, USA). *Lactobacillus casei* (BCC36987) purchased from Biotec, Thailand were prepared and cultured in de Man, Rogosa, and Sharpe (MRS) broth and agar (Difco^™^, USA). The bacteria were maintained in a 5% CO_2_ incubator at 37°C.

### Minimum Bactericidal Concentration

Minimum inhibitory concentration was determined by using broth microdilution assay. An aqueous stock solution of PG extract at 1000 mg/mL concentration was prepared and filtered. The PG extract mixture was prepared one step higher than the final dilution range required to compensate for the addition of an equal volume of inoculum. Two-fold serial dilutions of filtered PG extract were prepared with the appropriate broth medium specific for each of the bacterial strains at a total volume of 100 μL per well in 96-well polystyrene microtiter plates. The final concentrations of PG extract ranged decreasingly from 500 to 62.5 mg/mL. The microtiter plate wells were then inoculated with each bacterial strain separately.

After incubation at 37°C with 5% CO_2_ for 24 hours, 10 μL drop from each well were dispensed in selective culture agar for each bacteria and allowed to incubate for another 24 hours at the same conditions as previously done. The lowest concentration of antimicrobial agent that prevented the growth of an organism after subculture on to antibiotic-free media was considered to be the minimum bactericidal concentration (MBC). All experiments were conducted in triplicate.

### Time-kill Assay

About 500 mg/mL concentration of the PG extract was mixed with each bacteria separately. To determine the time-kill effects, 100 μL of mixture from each exposure time of 0, 1, 6, 8, 12, and 24 hour were taken, neutralized, and diluted 10-fold with phosphate buffer solution. In triplicate, 10 μL of the diluted samples were dispensed in selective culture media using the drop plate technique and incubated at 37°C with 5% CO_2_ for 24 hours. The total viable colonies (CFU/mL) were counted and log_10_ reductions were determined for each time point as compared to a negative control where no extract was added to the bacterial mixture.

### Agar Well Diffusion

Using the pour plate technique, 20 mL of Mueller-Hinton agar were seeded with 500 μL of *S. mutans* and was poured into sterile glass petri dishes and allowed to solidify completely. The same preparatory steps were done using MSA and MRS agar for *S. sanguinis,* and *L. casei* respectively. After the medium was solidified, 3 wells of 6 mm diameter × 6 mm height were made in each of the plates with the use of a sterile borer. One well was for the formulated PG gel, the other was for blank gel, and the other for the positive control 2% CHX gel. The plates were incubated overnight at 37°C in 5% CO_2_ condition. Inhibition of bacterial growth was determined by measuring the diameter of the zone of inhibition (ZOI) around each of the wells.

### Data Analysis

Statistical package for the social sciences version 19^®^ for Windows was used in data analysis. Descriptive statistics that included means, standard deviation, percentage (%), and log_10_ values were used. One way repeated measures analysis of variance (ANOVA) with *post hoc* Bonferroni test were used to compare the bacterial growth inhibition by PG gel, positive control 2% CHX gel, and blank gel in each of the bacterial strains. Comparison of bacterial growth inhibition by PG gel in the tested bacterial strains were done using one way ANOVA followed by *post hoc* Tamhane’s T2 test for multiple comparisons where equal variances were not assumed. Significance level is set at 0.05.

## RESULTS

### High Performance Liquid Chromatography

**Graphs 1A and B:**
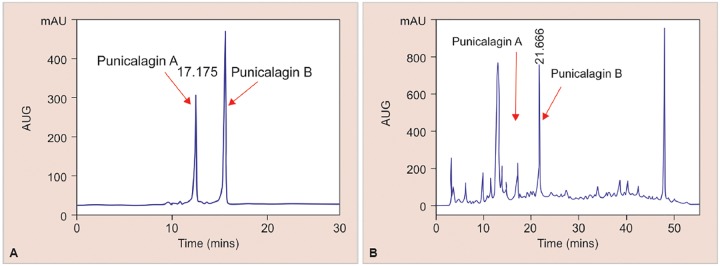
High Performance Liquid Chromatography chromatogram of 200 pg/mL punicalagin (A) and 50 mg/mL diluted aqueous PG extract sample; and (B) showing similar retention time of punicalagin A at the 17 minute range and punicalagin B at the 21 minute range

The HPLC method for determination of punicalagin content in the sample used in this study was successfully validated as summarized in Table 1. All parameters were within the acceptable limits.^20^ The peaks for punicalagins A and B were identified and quantified in the prepared 50 mg/mL diluted aqueous PG extract sample which were similar to the retention times of the peaks found in the 200 μg/mL punicalagin standard (Graphs 1A and B). Using the regression equation from the calibration curve, it was quantified that the total amount of punicalagin in the sample is an average of 2023.58 ± 25.29 μg/mL per 500 mg/mL aqueous PG extract.

**Table 1:** **Table 1:** Summary of the results for the linearity of standard and HPLC method validation for total punicalagin (punicalagin A + punicalagin B)

*Criteria*		*Obtained values*		*Acceptance criteria*	
Linearity range		25-200 μg/mL		N/A	
Regression equation		y = 152.8 x-2073		N/A	
Slope of calibration curve (m)		152.8		N/A	
Correlation coefficient (R^2^)		0.999		Not less	
				than 0.99	
Limit of detection		13.56 μg/mL		< 25 μg/mL	
Limit of quantification		13.57 μg/mL		< 25 μg/mL	
Precision		0.03-1.04%		≤ 2% RSD	
Accuracy		98.68-102.79%		95-105%	

N/A: Not applicable; RSD: Relative standard deviation

### Minimum Bactericidal Concentration

The MBC results for PG extract in distilled water showed inhibition of different bacteria at various concentrations. The MBC for the tested bacteria were the following: 250 mg/mL for *S. mutans,* 125 mg/mL for *S. sanguinis* and 500 mg/mL for *L. casei.*

### Time-kill Assay

**Graphs 2A to C:**
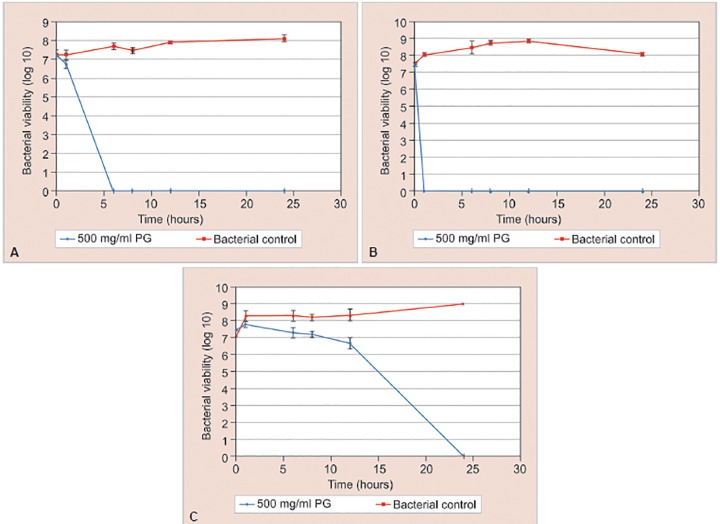
Time-kill curves for (A) S. *mutans;* (B) S. *sanguinis;* and (C) *L. casei* treated with 500 mg/mL aqueous PG extract. The graph is plotted as the logarithm of the number of remaining viable cells (log_10_ CFU/mL) against time

Results showed that for all three strains, 500 mg/mL aqueous PG extract equivalent to punicalagin 2023.58 ± 25.29 μg/mL can cause an increase in reduction of total viable colonies with time as compared to negative control (Graph 2). There was total bacterial growth inhibition of *S. mutans at* 6 hours (Graph 2A); *S. sanguinis* at 1 hour (Graph 2B) and *L. casei* at 24 hours (Graph 2C).

### Agar Well Diffusion

The results as shown in Table 2 revealed that for *S. mutans,* there is a significant difference in all three gels where the mean ZOI of CHX gel > PG gel > blank gel. For *S. sanguinis,* CHX gel = PG gel (p = 0.101) > blank gel. For *L. casei,* CHX gel > PG gel whereas PG gel = blank gel. Comparison of the PG gel potency against the tested bacteria showed that the mean ZOI in *S. sanguinis* = *S. mutans* (p = 0.118); *L. casei* < *S. mutans* and *S. sanguinis.*

**Table 2:** **Table 2:** Zones of inhibition of the three gels in each of the tested bacterial strains

		*Zones of Inhibition*					
Gel		S. *mutans Mean ± SD (mm)*		*S. sanguinis Mean ± SD (mm)*		*L. casei Mean ± SD (mm)*	
PG		16.72 + 2.32^b^		24.5 ± 7^b^		0	
CHX		31.18 ± 2.89^a,b^		26.66 ± 5.57^b^		22.88 ± 3.1^a,b^	
Blank		0		0		0	

SD: Standard deviation; Note: PG gel = 0.34% punicalagin (w/w); CHX gel = 2%; Level of significance set at p = 0.05; a = statistically significant higher mean ZOI than PG gel by means of one way repeated measures ANOVA (p < 0.001); b = statistically significant higher mean ZOI than blank gel by means of one way repeated measures ANOVA (p < 0.001)

## DISCUSSION

Crude PG extract cannot dissolve completely in most solvents, therefore, the identification and quantification of the bioactive ingredient in the sample is important. Focus was placed on punicalagin as the main active ingredient since it is completely soluble in water. In addition, the punicalagin used in this study was below the toxic level for punica-lagin recorded in literature.^14,18,21,22^ Several studies have concluded that the antimicrobial effect of pomegranate was due to the inhibitory activity exhibited by punicalagin against different tested bacteria and fungi.^18,22^

The results of the agar well diffusion experiment showed that the formulated PG gel exhibited a statistically significant greater inhibition in *S. mutans* than the negative control but at a significantly lesser inhibition than CHX. The PG gel may not be the gold standard in inhibiting *S. mutans,* however, it is able to reduce the growth of S. *mutans* significantly more than the negative control. Several studies have shown that PG extracted from different parts of the plant can inhibit *S. mutans* at various dilutions, such as 1:8 and 1:16 mg/mL with results comparable to the inhibition by CHX.^16,23^ In a recent *in vitro* study, pomegranate pulp gel showed highly significant inhibitory effect against *S. mutans* at concentrations ranging from 5 to 100% w/v.^24^ There was also another study which showed that 500 mg/mL aqueous PG extract presented with a mean ZOI of 23 mm.^25^ These experimental results from other studies supported the results of this study on *S. mutans* susceptibility to PG extract.

*Streptococcus sanguinis* was also found to be sensitive to pomegranate extract. The MBC of PG extract for *S. sanguinis* was 125 mg/mL which was the lowest among the three tested bacteria and required only 1 hour contact time at 4 × MBC (500 mg/mL) for complete inhibition of this bacterial strain. Agar well diffusion results showed that the formulated PG gel exhibited a zone of inhibition in *S. sanguinis* similar to the inhibition caused by CHX gel which is in agreement with previous studies that PG gel/extract at 1:8 and 1:16 mg/mL dilution presented an inhibitory activity on *S. sanguinis* in a similar or greater efficiency than the positive control.^16,23^ The previous studies were also in agreement with the results of this study that there was no difference in the effectiveness of PG in inhibiting the growth of *S. mutans* and *S. sanguinis.^16,23^*

For *L. casei,* the data gathered in the MBC and time-kill assay experiments showed that its growth can be completely inhibited by 500 mg/mL aqueous PG extract at 24 hours incubation time. However, results from the Agar well diffusion experiments have shown that the same concentration of PG in gel form cannot inhibit *L. casei* at 24 hour incubation period. These negative inhibitory results were in agreement with the results of some studies which concluded that lactobacilli, such as *L. casei* were generally not affected by ellagitannins (punicalagin) from commercially available pomegranate extract.^26,27^ This was further supported by studies which claimed that berries and their phenolic extracts contain organic acids and their addition to the culture media lowered the pH which favors the growth of Lactobacillus species.^26,28^ However, the results of the study by Devi claimed that the ZOI of 500 mg/mL PG in Lactobacillus is 19 mm which suggests that the aqueous PG extract does have an effect on *L. casei* inhibition.^25^ There is a need for further studies using increased concentrations of the PG gel as well as increased contact time to confirm the antibacterial activity of the formulated PG gel against *L. casei.*

The inhibitory activities on bacterial growth and adhesion can also be explained by the mechanism of action of PG extract. The tannins which are chemical compounds that precipitate protein, crosses over the cell wall of the microorganisms and binds to its surface leading to the precipitation of membrane proteins which can result in microbial cell lysis. The formation of complexes with cell wall proteins also decreases cell wall permeability and reduces the transport of substrates into the microbial cell. Tannins could also inhibit important bacterial enzymes, such as glucosyltransferase which is important for binding of the bacteria to the tooth surface as well as to each other and if disrupted, it prevents the adherence mechanism of these organisms to tooth surface.^12,26,29^ Another mechanism used by PG extract is the binding of phenolic compounds to carbohydrates and physiological metal ions, such as iron and copper making them unavailable for the microorganisms which affect the activity of metalloenzymes resulting in cell wall disruption. Polyphenols can also significantly affect the bacterial population by decreasing the pH of the environment.^12,26,29^ Although several studies have concluded that punicalagin is the major active constituent responsible for the antibacterial activity of PG, Lalwani et al stated that the synergistic effect of PG constituents is responsible for the anticariogenic effect of PG.^29^

## CONCLUSION

The PG gel tested in this study produced an inhibitory activity on *S. mutans* and *S. sanguinis* but not on *L. casei* within 24 hours incubation period. The findings of this study support that 500 mg/mL PG gel equivalent to 0.234% punicalagin (w/w) can possibly be used and is a potential candidate for the prevention of dental caries. Further *in vitro* biofilm and clinical research is needed to identify the real benefits of PG extract in gel form as a chemotherapeutic and preventive agent for dental caries.

## References

[1] Do LG (2012). Distribution of caries in children: variations between and within populations.. J Dent Res.

[2] Kassebaum NJ, Bernabe E, Dahiya M, Bhandari B, Murray CJ, Marcenes W (2015). Global burden of untreated caries: a systematic review and metaregression.. J Dent Res.

[3] Vahabi S, Najafi E, Alizadeh S (2011). *In vitro* antimicrobial effects of some herbal essences against oral pathogens.. J Med Plant Res.

[4] Kim JE, Kim HE, Hwang JK, Lee HJ, Kwon HK, Kim BI (2008). Antibacterial characteristics of *Curcuma xanthorrhiza* extract on *Streptococcus mutans* biofilm.. J Microbiol.

[5] Liljemark WF, Bloomquist C (1996). Human oral microbial ecology and dental caries and periodontal diseases.. Crit Rev Oral Biol Med.

[6] Callaway A, Kostrzewa M, Willershausen B, Schmidt F, Thiede B, Kupper H, Kneist S (2013). Identification of *Lactobacilli* from deep carious lesions by means of species-specific PCR and MALDI-TOF mass spectrometry.. Clin Lab.

[7] Svec P, Sedlacek I, Zackova L, Novakova D, Kukletova M (2009). *Lactobacillus* spp. associated with early childhood caries.. Folia Microbiol (Praha)..

[8] Tardif G, Sulavik MC, Jones GW, Clewell DB (1989). Spontaneous switching of the sucrose-promoted colony phenotype in *Streptococcus sanguinis.*. Infect Immun.

[9] Yamaguchi M, Terao Y, Ogawa T, Kawabata S (2006). Role of *Streptococcus sanguinis* sortase A in bacterial colonization.. Micro Infect.

[10] Devi A, Singh V, Bhatt AB (2011). Antibiotic sensitivity pattern of *Streptococcus* against commercially available drugs and comparison with extract of *Punica granatum.*. Int J Pharm Sci Res.

[11] Twetman S (2010). Antibacterial agents for prevention and therapy of early childhood caries.. Oralprophylaxe Kinderzahnheilkunde.

[12] Ismail T, Sestili P, Akhtar S (2012). Pomegranate peel and fruit extracts: a review of potential anti-inflammatory and anti-infective effects.. J Ethnopharmacol.

[13] Gaig P, Bartolome B, Lleonart R, Garcia-Ortega P, Palacios R, Richart C (1999). Allergy to pomegranate *(Punica granatum).*. Allergy.

[14] Patel C, Dadhaniya P, Hingorani L, Soni MG (2008). Safety assessment of pomegranate fruit extract: acute and subchronic toxicity studies.. Food Chem Toxicol.

[15] Vidal A, Fallarero A, Pena BR, Medina ME, Gra B, Rivera F, Gutierrez Y, Vuorela PM (2003). Studies on the toxicity of *Punica gra-natum* L. (Punicaceae) whole fruit extracts.. J Ethnopharmacol.

[16] Vasconcelos LC, Sampaio FC, Sampaio MC, Pereira Mdo S, Higino JS, Peixoto MH (2006). Minimum inhibitory concentration of adherence of *Punica granatum* Linn (pomegranate) gel against *S. mutans, S. mitis* and *C. albicans.*. Braz Dent J.

[17] Machado TB, Pinto AV, Pinto MC, Leal IC, Silva MG, Amaral AC, Kuster RM, Netto-dos Santos KR (2003). *In vitro* activity of Brazilian medicinal plants, naturally occurring naphthoquinones and their analogues, against methicillin-resistant *Staphylococcus*
*aureus.*. Int J Antimicrob Agents.

[18] Abdollahzadeh Sh, Mashouf R, Mortazavi H, Moghaddam M, Roozbahani N, Vahedi M (2011). Antibacterial and antifungal activities of *Punica granatum* peel extracts against oral pathogens.. J Dent (Tehran).

[19] Madrigal-Carballo S, Rodriguez G, Krueger CG, Dreher M, Reed JD (2009). Pomegranate *(Punica granatum)* supplements: authenticity, antioxidant and polyphenol composition.. J Funct Foods.

[20] Goud MV, Rao AS, Ranjan SP, Shalini SD, Sowmya S, Bhoga B (2013). method development and validation of RP-HPLC method for assay of sildosin in pharmaceutical dosage form.. Int J Pharma Sci.

[21] Cerda B, Llorach R, Ceron JJ, Espin JC, Tomas-Barberan FA (2003). Evaluation of the bioavailability and metabolism in the rat of punicalagin, an antioxidant polyphenol from pomegranate juice.. Eur J Nutr.

[22] Machado TB, Leal ICR, Amaral ACF, dos Santos KRN, da Silva MG, Kuster RM (2002). Antimicrobial ellagitannin of *punica granatum* fruits.. J Braz Chem Soc.

[23] Pereira JV, Pereira MSV, Sampaio FC, Sampaio MCC, Alves PM, de Araujo CRF, Higino JS (2006). In vitro antibacterial and antiadherence effect of *Punica granatum* Linn extract upon dental biofilm microorganisms.. Rev Bras Farmacogn.

[24] Subramaniam P, Dwivedi S, Uma E, Girish Babu KL (2012). Effect of pomegranate and *Aloe vera* extract on *Streptococcus mutans:* An *in vitro* study.. Dent Hypotheses.

[25] Devi A, Singh V, Bhatt AB (2011). Comparative antibacterial study of different extract of pomegranate and its wild variety.. Int J Pharm Sci Res..

[26] Bialonska D, Kasimsetty SG, Schrader KK, Ferreira D (2009). The effect of pomegranate *(Punica granatum L.)* byproducts and ellagitannins on the growth of human gut bacteria.. J Agr Food Chem.

[27] Landete JM (2011). Ellagitannins, ellagic acid and theri derived metabolites: A review about source, metabolism, functions and health.. Food Res Int.

[28] Puupponen-Pimia R, Nohynek L, Alakomi HL, Oksman-Caldentey KM (2005). Bioactive berry compounds—novel tools against humanpathogens.. Appl Microbiol Biotechnol.

[29] Lalwani V, Koneru A, Vanishree M, Vardendra M, Hunasgi S, Surekha R (2014). Anti-microbial activity of *Punica granatum* on *Streptococcus* in dental caries patients and healthy individuals: a comparative study.. J Adv Clin Res In.

